# Detection of nephrocalcinosis using ultrasonography, micro‐computed tomography, and histopathology in cats

**DOI:** 10.1111/jvim.17011

**Published:** 2024-02-13

**Authors:** Pak‐Kan Tang, Rebecca F. Geddes, Yu‐Mei Chang, Rosanne E. Jepson, Dirk Hendrik Nicolaas van den Broek, Nicola Lötter, Jonathan Elliott

**Affiliations:** ^1^ Department of Comparative Biomedical Sciences, Royal Veterinary College University of London London United Kingdom; ^2^ Department of Clinical Science and Services, Royal Veterinary College University of London London United Kingdom; ^3^ Research Support Office, Royal Veterinary College University of London London United Kingdom; ^4^ Department of Clinical Sciences, Faculty of Veterinary Medicine Utrecht University Utrecht The Netherlands

**Keywords:** calcification, chronic kidney disease, CKD‐MBD, feline, mineralization

## Abstract

**Background:**

Identification of nephrocalcinosis in cats with chronic kidney disease (CKD) is of clinical interest but the ability of ultrasonography to detect nephrocalcinosis is uncertain.

**Objectives:**

To compare ultrasonography, micro‐computed tomography (μCT) and histopathology for identification of nephrocalcinosis.

**Animals:**

Twelve kidneys from 7 euthyroid client‐owned cats with CKD.

**Methods:**

Descriptive study. Renal ultrasonography was performed ante‐mortem for nephrocalcinosis detection. Kidneys were grouped based on nephrocalcinosis: present, suspected, or absent. When cats died, necropsy was performed. Renal tissue was evaluated using μCT for macroscopic nephrocalcinosis, and nephrocalcinosis volume‐to‐kidney tissue ratio (macro‐VN:KT) and sagittal nephrocalcinosis area‐to‐kidney tissue ratio (macro‐AN:KT) were calculated. Each kidney subsequently was bisected longitudinally, formalin‐fixed, and paraffin‐embedded for microscopic nephrocalcinosis assessment using von Kossa and Alizarin red staining with AN:KT (VK‐micro‐AN:KT and AR‐micro‐AN:KT) quantified using ImageJ. Data are presented as median (range). Relationships between macroscopic and microscopic AN:KT were assessed using Spearman's correlation.

**Results:**

Nephrocalcinosis by ultrasonography was considered to be absent in 3, suspected in 3, and present in 5 kidneys; 1 kidney had nephrolithiasis with nephrocalcinosis. The macro‐VN:KT was 0.001%, 0.001%, and 0.019%, and the macro‐AN:KT was 0.08%, 0.30%, and 1.47%, respectively. Histologically, VK‐micro‐AN:KT was 0.21%, 2.85%, and 4.56%, and AR‐micro‐AN:KT was 1.73%, 5.82%, and 8.90% for kidneys where ultrasonographic macro‐nephrocalcinosis was absent, suspected, or present, respectively. A strong correlation was identified between macroscopic (macro‐AN:KT) and microscopic (VK‐micro‐AN:KT) nephrocalcinosis (*r*_s_ = 0.76; *P* = .01).

**Conclusions and Clinical Importance:**

Ultrasonographically diagnosed nephrocalcinosis correlates well with macroscopic and microscopic nephrocalcinosis at necropsy despite their separation in time.

**Abbreviations**
AR‐micro‐AN:KT: Alizarin red‐stained nephrocalcinosis area‐to‐kidney tissue ratio
CKD: chronic kidney disease
FFPE: formalin‐fixed paraffin‐embedded
IRIS: International Renal Interest Society
macro‐AN:KT: nephrocalcinosis area‐to‐kidney tissue ratio
macro‐VN:KT: nephrocalcinosis volume‐to‐kidney tissue ratio
SDMA: symmetric dimethylarginine
TT4: total thyroxine
USG: urine specific gravity
UTI: urinary tract infection
VK‐micro‐AN:KT: von Kossa‐stained nephrocalcinosis area‐to‐kidney tissue ratio
μCT: micro‐computed tomography

## 1 INTRODUCTION

Nephrocalcinosis is characterized by the deposition of calcium phosphate or calcium oxalate crystals in the kidneys.^1^ Visualization of nephrocalcinosis without further magnification is termed macroscopic nephrocalcinosis and ultrasonography is suggested to be the preferred imaging modality for visualization in human patients.^2^, ^3^ Nephrocalcinosis is prevalent in humans with chronic kidney disease (CKD), especially in dialysis patients with end‐stage renal disease.^4^, ^5^ Serum calcium concentration was found to be an independent risk factor for microscopic nephrocalcinosis in humans with CKD,^5^ and its occurrence (both microscopic and macroscopic) is suggested to be associated with accelerated deterioration of renal function.^6^, ^7^, ^8^, ^9^ Although microscopic nephrocalcinosis previously has been identified in cats with CKD, with a prevalence of 50%‐58% and 78% documented at necropsy in 2 different retrospective studies,^10^, ^11^ reports on the detection of macroscopic nephrocalcinosis (referred to herein as nephrocalcinosis) using ultrasonography in cats are scarce. A recent study reported that a significantly higher proportion of azotemic cats (any cause) have ultrasonographic evidence of nephrocalcinosis compared to non‐azotemic cats, but the proportion in both groups was low, with only 5% (12/238) and 1% (3/270) documented, respectively.^12^ This observation suggests that ultrasonography underestimates the detection of nephrocalcinosis in cats, although histological analysis of these kidneys was not available for comparison, and the majority of these cats did not have a final diagnosis reported. Anecdotally, nephrocalcinosis can be visualized frequently on ultrasonography in cats with CKD. Therefore, our objective was to compare ultrasonography, micro‐computed tomography (μCT) and histopathology for the identification of nephrocalcinosis in cats with azotemic CKD.

## 2 METHODS

### 2.1 Case selection

Azotemic CKD cats that had renal ultrasonography performed as part of a larger prospective longitudinal study with kidney tissues available from necropsy were selected for study. Informed consent was obtained from the owner and approval of the Royal Veterinary College Ethics and Welfare Committee (URN20131258E) was granted. A diagnosis of azotemic CKD was defined as a plasma creatinine concentration ≥2 mg/dL with a urine specific gravity (USG) <1.035, or plasma creatinine concentration ≥2 mg/dL on 2 consecutive occasions without evidence of a pre‐renal cause. Blood samples were collected by jugular venipuncture into heparinized tubes, and urine samples were obtained by cystocentesis. Samples were stored at 4°C for <6 hours before centrifugation and separation. Heparinized plasma was analyzed biochemically at an external laboratory (IDEXX laboratories, Wetherby, UK). In‐house urinalyses, including USG measurement by refractometry, dipstick chemical analysis and microscopic urine sediment examination, were performed on the day of collection. All cats with a confirmed diagnosis of CKD had been offered a phosphate‐restricted diet. Cats were excluded if they had clinically suspected hyperthyroidism with plasma total thyroxine (TT4) concentration >40 nmol/L or if they were receiving medical management for hyperthyroidism, diabetes mellitus or recurrent urinary tract infection (UTI). Cats also were excluded if they were fed a veterinary diet to manage urolithiasis, or were being treated with corticosteroids, furosemide, bisphosphonates or calcium‐based phosphate binders. Cats receiving amlodipine besylate for systemic hypertension were included. Cats that could not be scanned without sedation or anesthesia were excluded.

### 2.2 Renal ultrasonography

Renal ultrasound examinations were performed by the same operator (Dr. P‐K Tang) using a 12L‐RS linear transducer with a 5 to 13 MHz frequency range (Logiq e R7 console, GE HealthCare, Buckinghamshire, UK). Cats were placed in lateral recumbency. The abdomen was clipped and acoustic coupling gel applied before scanning. All images were saved in digital imaging and communications in medicine (DICOM) format. For each kidney, both sagittal and transverse planes were evaluated, with color Doppler used to identify the arcuate blood vessels for locating the corticomedullary junction, especially in kidneys with poor corticomedullary differentiation. All ultrasound images and cine loop clips captured were blindly reviewed by the same residency‐trained radiologist (Dr. D.H.N van den Broek) for the detection of nephrocalcinosis. Nephrocalcinosis was defined as the identification of calcification within the renal parenchyma, outside the collecting system.^13^, ^14^ Nephrolithiasis was defined as the identification of calcification within the collecting system.^2^ Renal mineralization (both nephrocalcinosis and nephrolithiasis) was identified by the presence of a hyperechoic area with acoustic shadowing.^15^ In conditions where a hyperechoic area was identified but no acoustic shadowing was evident, presence of renal mineralization was classified as “suspected.”

### 2.3 Micro‐computed tomography for macroscopic nephrocalcinosis

At necropsy, kidneys, either fresh or fixed in 10% neutral buffered formalin for <24 hours, were scanned for macroscopic nephrocalcinosis using a μCT scanner (Skyscan 1172, Bruker, Kontich, Belgium) and small (4000 × 2672 pixels) camera without a filter. Images were obtained using the following scanning parameters: isotropic voxel size 5 μm per pixel, source voltage 70 kV, source current 140 μA, exposure time 670 ms, and imaging rotation scan 180° with a 0.4° rotation step. Projection images were reconstructed into tomograms using NRecon 1.7.5.9 (Bruker, Kontich, Belgium) and repositioned using Dataviewer 1.5.6.6 (Bruker, Kontich, Belgium). Tomograms were analyzed using Bruker analysis software CTAn 1.20.3 (Bruker, Kontich, Belgium) and volume rendered 3‐dimensional visualizations were created using CTVox 3.3 (Bruker Kontich, Belgium). For each kidney, tissue volume and nephrocalcinosis volume were calculated using the 3‐dimensional analysis tool in CTAn, with threshold levels set at 0 to 30 and 0 to 140, respectively. The macroscopic nephrocalcinosis volume‐to‐kidney tissue ratio (macro‐VN:KT) was calculated using the formula:

$$\mathrm{VN}:\mathrm{KT}\;\left(\%\right)=\frac{\text{Volume of nephrocalcinosis}}{\text{Volume of kidney tissue}}\times 100\%.$$

A sagittal view of each kidney as a single frame was created on CTVox. Tissue area and nephrocalcinosis area were calculated using CTAn, with threshold levels set at 0 to 30 and 0 to 100, respectively. The macroscopic nephrocalcinosis area‐to‐kidney tissue ratio (macro‐AN:KT) was calculated using the formula:

$$\text{AN}:\mathrm{KT}\;\left(\%\right)=\frac{\text{Area of nephrocalcinosis}}{\text{Area of kidney tissue}}\times 100\%.$$

### 2.4 Histopathological data

Formalin‐fixed renal tissues were paraffin‐embedded (FFPE) for histopathological evaluation. A 4 μm section of FFPE tissue was stained using von Kossa's method to identify hydroxyapatite deposits.^16^ An adjacent FFPE section from each case was stained with Alizarin red solution (adjusted to pH 4.2 using 10% ammonium hydroxide). From each kidney, 2 stained sagittal kidney slides (von Kossa and Alizarin red) were digitally scanned. Open‐source software, ImageJ 2.9.0 (National Institutes of Health, Bethesda, Maryland), was used to quantify microscopic nephrocalcinosis. In brief, all images were converted to 8‐bit grayscale for thresholding. Quantification of the area of kidney tissue and the area of positively stained tissue (microscopic nephrocalcinosis) was performed using respective threshold levels of 0 to 230 and 0 to 170‐190 for the von Kossa‐stained images (VK‐micro‐AN:KT), and threshold levels of 0 to 230 and 0 to 165, respectively, for the Alizarin red‐stained images (AR‐micro‐AN:KT). The threshold level set for identifying mineralization was adjusted for each individual kidney because of variation in the intensity of stain uptake as a result of the different sizes of kidney tissue samples.

### 2.5 Statistical analysis

Descriptive statistics were performed because of limited sample size among the groups on the basis of nephrocalcinosis: present, suspected, or absent. The normality of continuous variables was assessed by visual inspection of Q‐Q plots and using the Shapiro‐Wilk test. Most data were not normally distributed and therefore numerical data are presented as median (25th, 75th percentile) for consistency. Relationships among macroscopic nephrocalcinosis volume (as determined by macro‐VN:KT), macroscopic nephrocalcinosis area (as determined by macro‐AN:KT) and microscopic nephrocalcinosis area (as determined by VK‐micro‐AN:KT and AR‐micro‐AN:KT) were assessed using Spearman's rank correlation.

## 3 RESULTS

**FIGURE 1:**
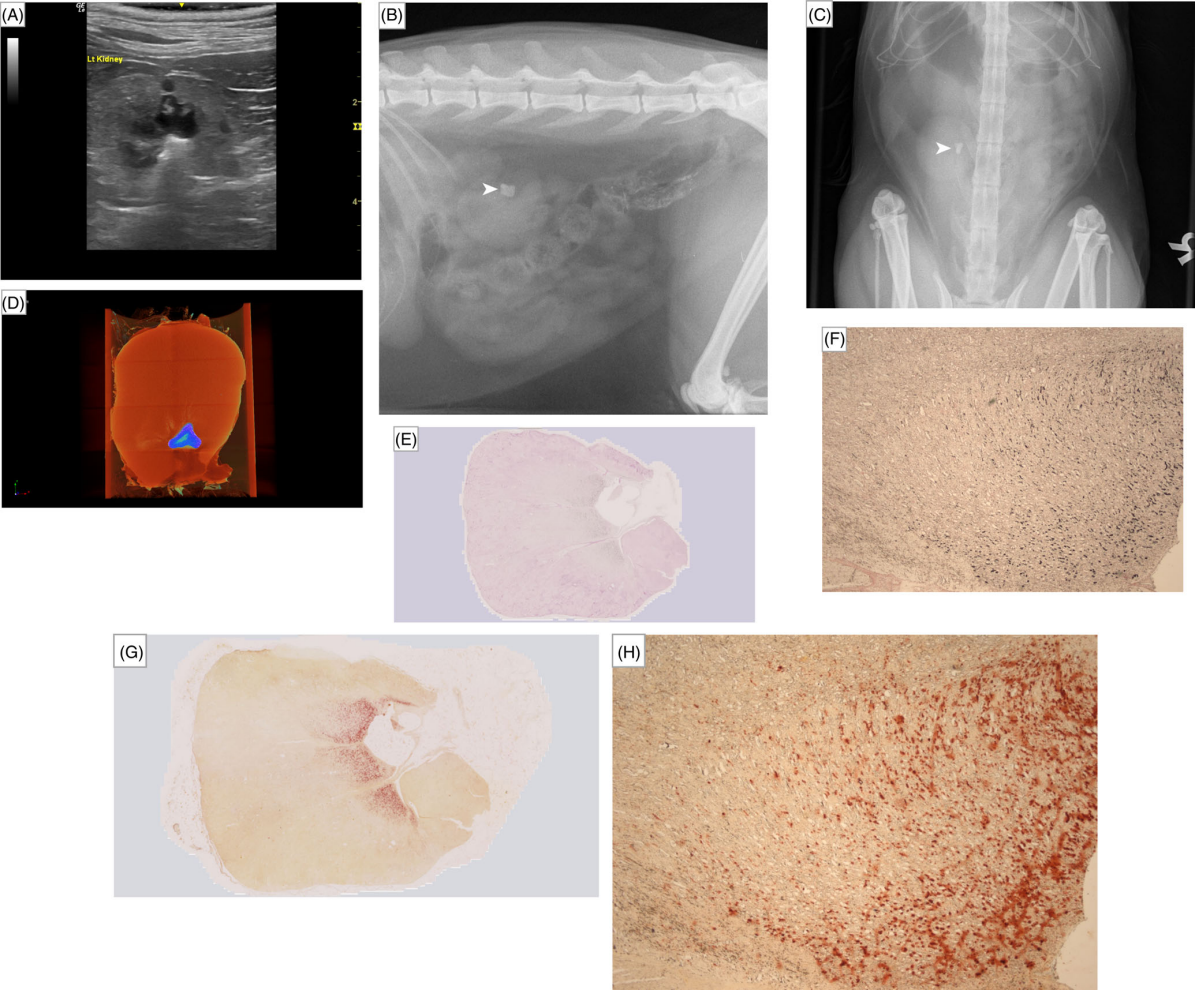
Images of the kidney with nephrolithiasis. (A) Sonographic image illustrating a hyperechoic interface with distal acoustic shadowing within the collecting system, together with mild renal pelvic dilatation, indicating the presence of nephrolithiasis. (B) Right lateral and (C) dorsoventral projections of abdominal radiographic images illustrating the presence of nephrolithiasis (white arrowhead) in the same kidney. (D) Micro‐computed tomography (μCT) image of the same kidney at necropsy, projection images were reconstructed into tomograms and volume‐rendered 3‐dimensional visualizations were created using Bruker μCT SkyScan software (NRecon and CTVox, respectively). Nephrolithiasis is highlighted in blue‐green coloring. Overview and magnified (2.5×) images of the kidney section after removal of the nephrolith, with nephrocalcinosis stained with (E and F) von Kossa and (G and H) Alizarin red; positive staining for calcium deposition are indicated by black and red coloring, respectively.

Twelve kidneys, obtained from 7 cats with CKD that underwent necropsy were included (2 kidneys had to be prepared differently for cell culture upon collection for a different study). Of the 7 cats with CKD, 6 were domestic shorthair and 1 was a Bengal. At the time the last ante‐mortem ultrasonography was performed, 4 cats had International Renal Interest Society (IRIS) stage 2, 1 had IRIS Stage 3 and 2 had IRIS stage 4 CKD. The median plasma concentrations of creatinine, symmetric dimethylarginine (SDMA), phosphate, total calcium and ionized calcium were 2.6 mg/dL (range, 2‐6.9 mg/dL), 20 μg/dL (range, 14‐41 μg/dL), 4.67 mg/dL (range, 3.44‐7.31 mg/dL), 10.56 (range, 9.48‐12.8 mg/dL), and 5.44 (range, 4.96‐6.32 mg/dL), respectively. Of the 12 kidneys, nephrocalcinosis detected by ultrasonography (ante‐mortem) was considered to be present in 5 kidneys, suspected in 3 and absent in 3. None of these cats had evidence of ureteral dilatation. One kidney had nephrolithiasis, which precluded accurate assessment of concurrent nephrocalcinosis on ultrasonography (Figure 1A) and therefore was not included in statistical analyses. Nephrolithiasis in this case was further confirmed using conventional radiography (Figure 1B,C) as well as μCT (Figure 1D) at necropsy. Quantitative mineral analysis of the urolith was performed at an external laboratory (Minnesota Urolith Centre, Saint Paul, Minnesota, USA) using optical crystallography and infrared spectroscopy^17^; the nephrolith was 100% calcium oxalate monohydrate. The time between last ante‐mortem ultrasonography and necropsy for all cats was a median of 167 days (range, 8‐255 days). At the visit nearest to death or euthanasia with a median duration of 50 days (range, 8‐157 days), the median plasma creatinine concentration from these 7 CKD cats was 2.6 mg/dL (range, 1.5‐7.7 mg/dL), with no change in their IRIS stages. The median plasma concentrations of SDMA, phosphate, total calcium and ionized calcium were 24 μg/dL (range, 14‐47 μg/dL), 5.17 mg/dL (4.21‐6.9 mg/dL), 10.88 mg/dL (range, 7.84‐12.08 mg/dL), and 5.44 mg/dL (range, 4.68‐5.84 mg/dL), respectively. Line graphs illustrating the changes in these clinicopathological variables in the 7 cats are presented in Figure S1.

**FIGURE 2:**
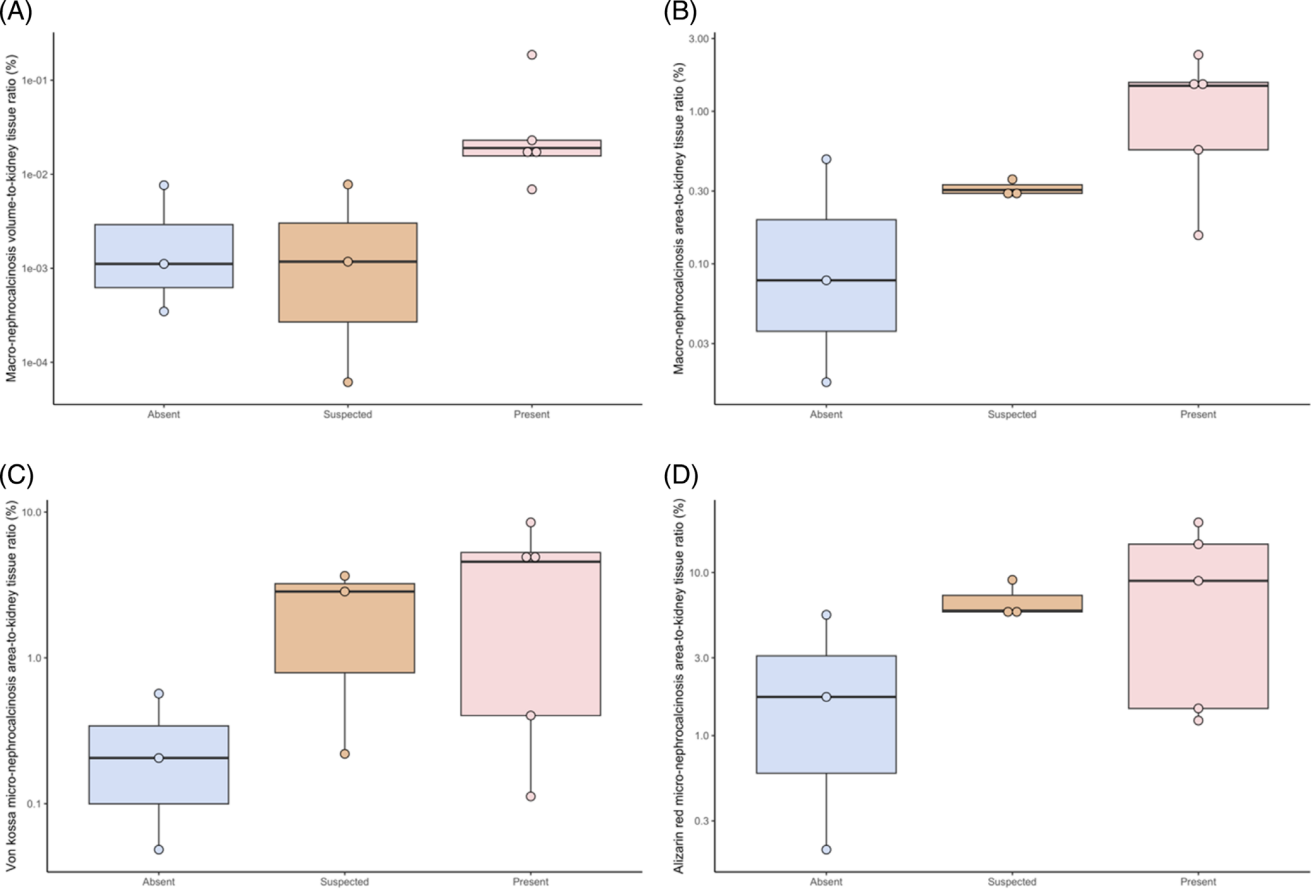
Boxplots illustrating the (A) macroscopic nephrocalcinosis volume‐to‐kidney ratio (macro‐VK:KT) and (B) macroscopic nephrocalcinosis area‐to‐kidney ratio (macro‐AN:KT), as determined by micro‐computed tomography (μCT), and (C) microscopic nephrocalcinosis area‐to‐kidney ratio from von Kossa stained sagittal kidney slides (VK‐micro‐AN:KT) and (D) Alizarin red stained sagittal kidney slides (AR‐micro‐AN:KT) according to the classification of nephrocalcinosis determined by ultrasonography at enrollment (“absent” vs “suspected” vs “present”) in 11 kidneys.

**FIGURE 3:**
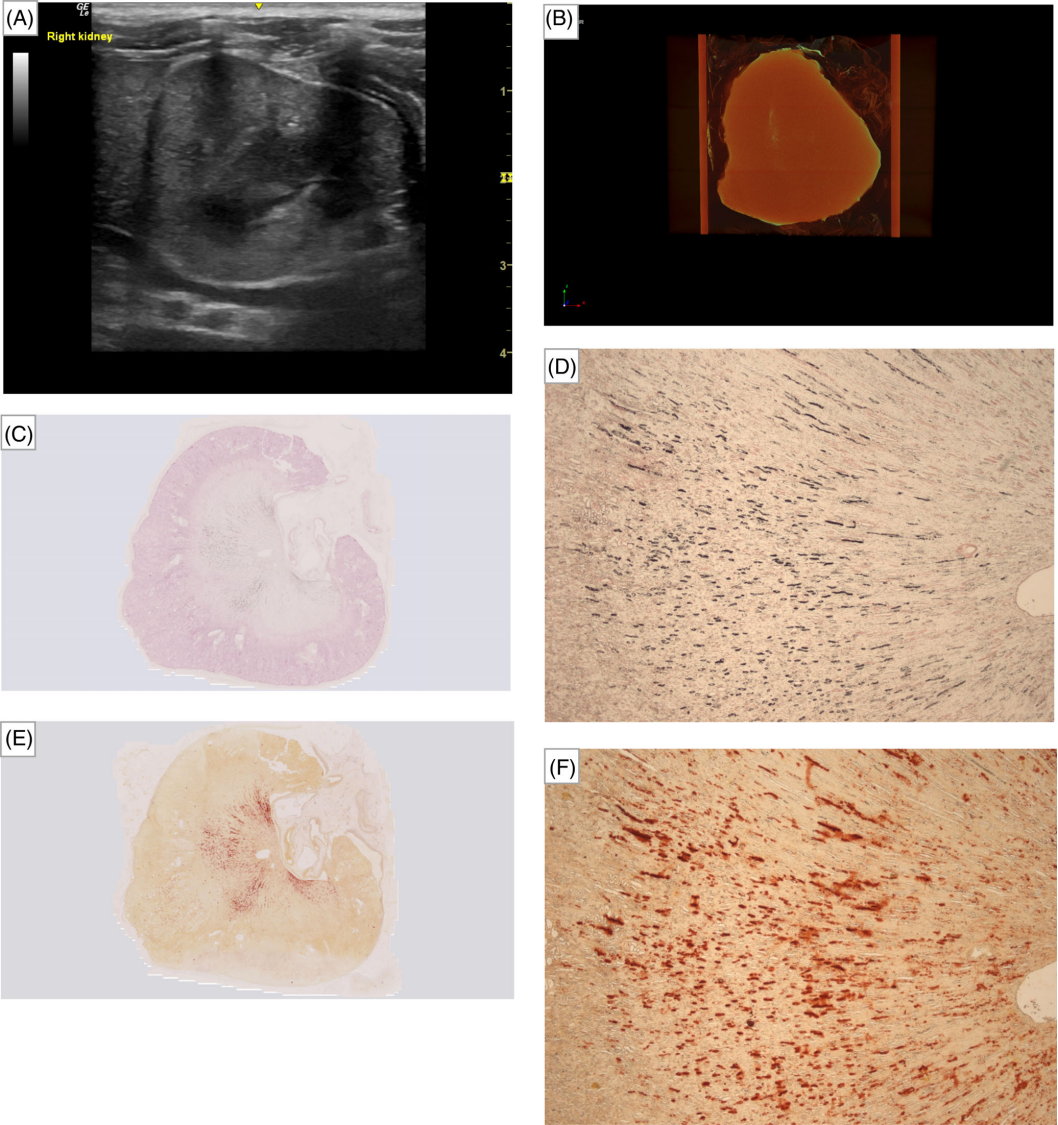
Collection of images of the kidney with absence of ultrasound‐diagnosed nephrocalcinosis. (A) Sonographic image illustrating no hyperechoic interface, indicating the absence of macroscopic nephrocalcinosis. (B) Micro‐computed tomography (μCT) image of the same kidney, projection images were reconstructed into tomograms and volume‐rendered 3‐dimensional visualizations were created using Bruker μCT SkyScan software (NRecon and CTVox, respectively). Macroscopic nephrocalcinosis is outlined in yellow‐green‐blue coloring. Overview and magnified (2.5×) images of the kidney section with nephrocalcinosis stained with (C and D) von Kossa and (E and F) Alizarin red; positive staining for calcium deposition are indicated by black and red coloring, respectively.

**FIGURE 4:**
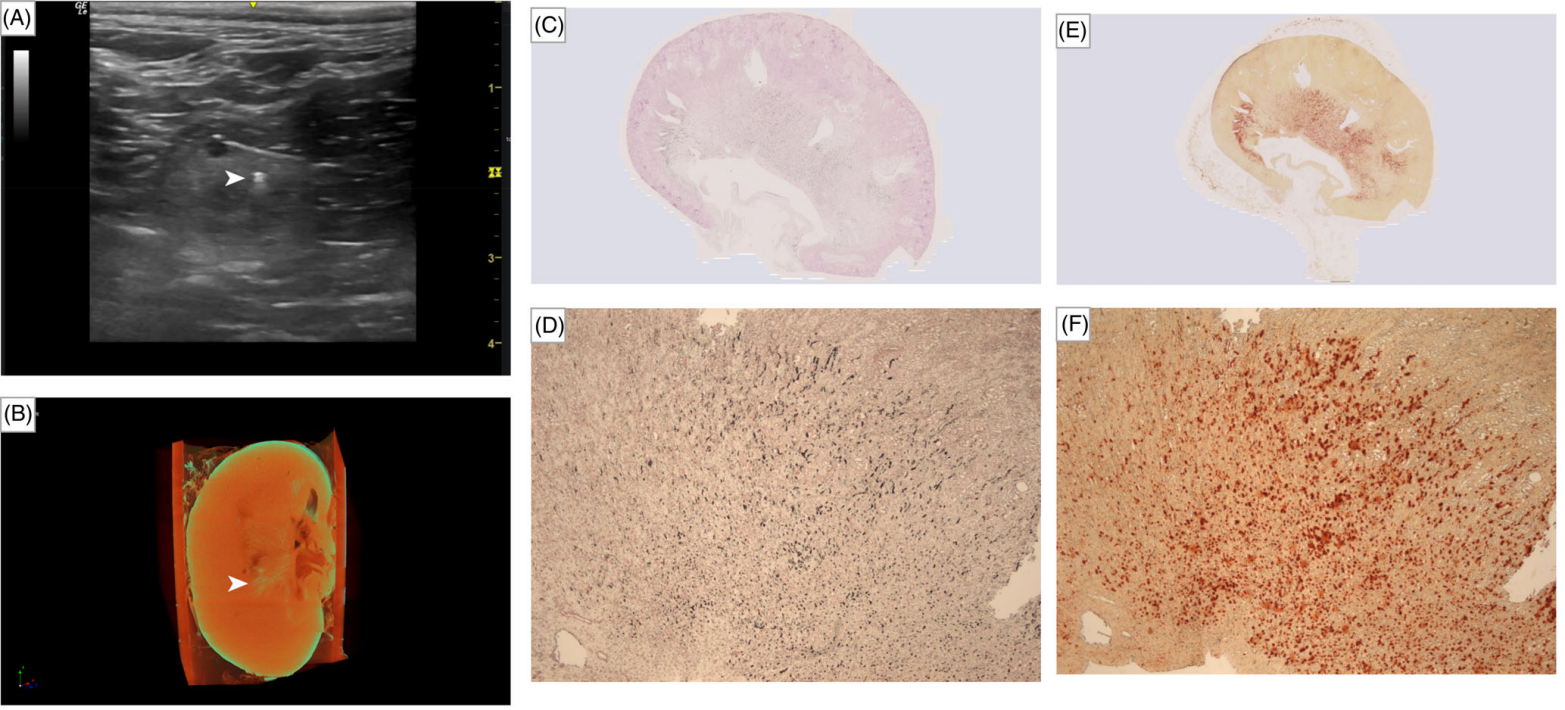
Collection of images of the kidney with suspected presence of ultrasound‐diagnosed nephrocalcinosis. (A) Sonographic image illustrating a hyperechoic interface without obvious distal acoustic shadowing (white arrowhead), suspicious of the presence of macroscopic nephrocalcinosis. (B) Micro‐computed tomography (μCT) image of the same kidney, projection images were reconstructed into tomograms and volume‐rendered 3‐dimensional visualizations were created using Bruker μCT SkyScan software (NRecon and CTVox, respectively). Macroscopic nephrocalcinosis is outlined in yellow‐green‐blue coloring (white arrowhead). Overview and magnified (2.5×) images of the kidney section with suspected nephrocalcinosis stained with (C and D) von Kossa and (E and F) Alizarin red; positive staining for calcium deposition are indicated by black and red coloring, respectively.

**FIGURE 5:**
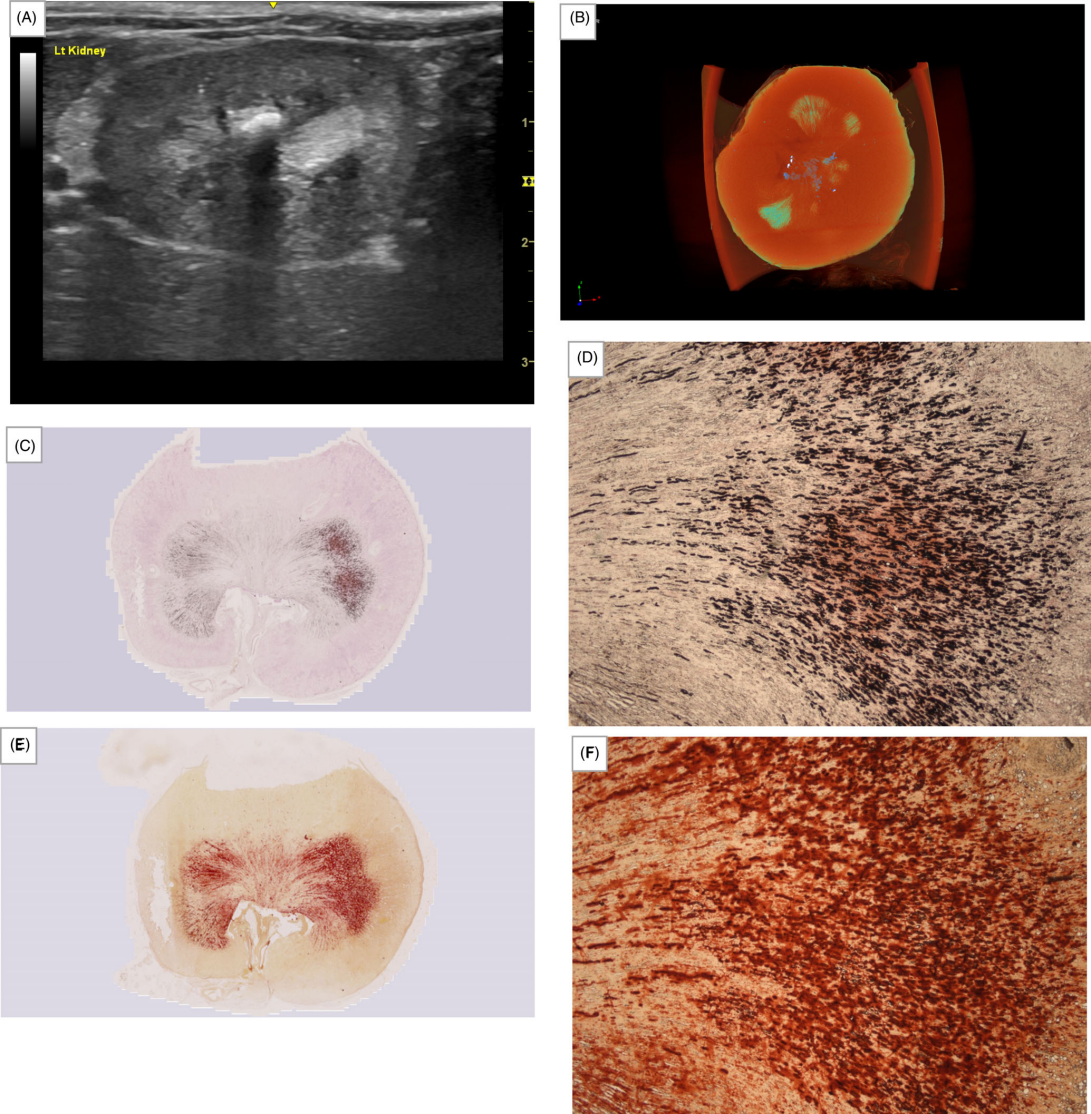
Collection of images of the kidney with presence of ultrasound‐diagnosed nephrocalcinosis. (A) Sonographic image illustrating a hyperechoic interface with distal acoustic shadowing, indicating the presence of macroscopic nephrocalcinosis. (B) Micro‐computed tomography (μCT) image of the same kidney, projection images were reconstructed into tomograms and volume‐rendered 3‐dimensional visualizations were created using Bruker μCT SkyScan software (NRecon and CTVox, respectively). Macroscopic nephrocalcinosis is outlined in yellow‐green‐blue coloring. Overview and magnified (2.5×) images of the kidney section with nephrocalcinosis stained with (C and D) von Kossa and (E and F) Alizarin red; positive staining for calcium deposition are indicated by black and red coloring, respectively.

The macro‐VN:KT, macro‐AN:KT, VK‐micro‐AN:KT, and AR‐micro‐AN:KT among the 3 nephrocalcinosis groups (n = 11) are presented in Figure 2, with graphically increasing severity of both macroscopic and microscopic nephrocalcinosis observed in cats with CKD with absence, suspected, and presence of ultrasound‐diagnosed nephrocalcinosis. A summary of their correlations is presented in Table 1. A strong positive correlation was identified between macroscopic (macro‐AN:KT) and microscopic (VK‐micro‐AN:KT) nephrocalcinosis (*r*_s_ = 0.76; *P* = .01). Moderate and excellent positive correlations were found between the area and volume of macroscopic nephrocalcinosis (*r*_s_ = 0.65; *P* = .04), and between the 2 staining techniques for microscopic nephrocalcinosis (*r*_s_ = 0.92; *P* = .001), respectively. Examples of sonographic, μCT and histopathologic images of each kidney with absence (Figure 3), suspected (Figure 4), and presence (Figure 5) of ultrasound‐diagnosed nephrocalcinosis are presented.

**TABLE 1:** Correlations between macroscopic and microscopic nephrocalcinosis in 11 kidneys from 7 cats with chronic kidney disease.

	macro‐AN:KT	macro‐VN:KT	AR‐micro‐AN:KT	VK‐micro‐AN:KT
macro‐AN:KT	1	***r***_**s**_ **= 0.65; *P* = .04**	*r*_s_ = .55; *P* = .09	***r***_**s**_ **= 0.76; *P* = .01**
macro‐VN:KT	***r***_**s**_ **= 0.65; *P* = .04**	1	*r*_s_ = 0.18; *P* = .60	*r*_s_ = 0.31; *P* = .34
AR‐micro‐AN:KT	*r*_s_ = 0.55; *P* = .09	*r*_s_ = 0.18; *P* = .60	1	***r***_**s**_ **= 0.92; *P* = .001**
VK‐micro‐AN:KT	***r***_**s**_ **= 0.76; *P* = .01**	*r*_s_ = 0.31; *P* = .36	***r***_**s**_ **= 0.92; *P* = .001**	1

*Note*: Significant correlations (*P* ≤ .05) are highlighted in bold.

Abbreviations: AR‐micro‐AN:KT, Alizarin red stained micro‐nephrocalcinosis area‐to‐kidney ratio; macro‐AN‐KT, macro‐nephrocalcinosis area‐to‐kidney ratio; macro‐VN‐KT, macro‐nephrocalcinosis volume‐to‐kidney ratio; VK‐micro‐AN:KT, von Kossa stained micro‐nephrocalcinosis area‐to‐kidney ratio.

## 4 DISCUSSION

We found a strong positive correlation between macroscopic and microscopic nephrocalcinosis at necropsy in cats with azotemic CKD. Ante‐mortem ultrasonographic detection of nephrocalcinosis also appears to correlate well with findings from μCT and histopathology after von Kossa and Alizarin red staining. High‐resolution X‐ray μCT is an emerging and sensitive 3‐dimensional tool for the visualization and quantification of vascular calcification in animal models.^18^, ^19^, ^20^ To our knowledge, this novel technique of utilizing μCT to evaluate the proportional volume and area of nephrocalcinosis in the entirety of necropsied feline kidneys has not been described previously.

Our results reinforced the high specificity of ultrasonography in the detection of nephrocalcinosis in feline kidneys. In a recent study in humans, ultrasonography was found to be superior to CT for detection of nephrocalcinosis.^2^ However, the sensitivity and specificity of such detection may be dependent on the operator performing the ultrasonography. All ultrasonographic images and cine loop clips captured in our study were blindly reviewed by the same residency‐trained radiologist to minimize potential misclassification bias.

We found excellent correlation between the 2 staining techniques used for the detection of calcium deposition histologically. This result is in agreement with a previous retrospective study.^11^ The principle of von Kossa staining is based on the transformation of calcium salts (eg, calcium phosphate, calcium oxalate) to silver salts, visualized as black metallic silver staining.^21^, ^22^ Another histochemical stain to identify the deposition of calcium crystals used in our study was Alizarin red, which binds directly to calcium ions.^23^ Alizarin red can be used to help distinguish calcium phosphate from calcium oxalate based on the pH applied.^24^ The pH of 4.2 used in our study does not highlight calcium oxalate because calcium oxalate can only be stained with Alizarin red at a pH of 7.^25^ Our results therefore indicate that nephrocalcinosis in feline kidneys is primarily composed of calcium phosphate crystals. This finding corroborates the results of previous studies involving experimental rats,^26^ human patients^5^ and cats with CKD.^11^

Nephrolithiasis was present in 1 kidney, and calculus analysis confirmed the nephrolith to be 100% calcium oxalate monohydrate, consistent with the most common composition for upper urinary tract uroliths in cats.^27^, ^28^ This kidney had exceptionally high macro‐AN:KT and macro‐VN:KT measured from μCT because of the presence of the nephrolith. However, tissue sectioning for histopathology could only be performed after removal of the nephrolith. Therefore, a decision to exclude the kidney with nephrolithiasis from all analyses in our study was made. Interestingly, this kidney did not appear to have a high extent of nephrocalcinosis as indicated by the results obtained using μCT (Figure 1D) and histopathology after von Kossa (Figure 1E,F) and Alizarin red staining (Figure 1G,H) after the removal of the nephrolith. Distinct pathophysiological mechanisms may exist between the development of nephrocalcinosis and nephrolithiasis in cats, despite their intimate relationship and common risk factors (eg, hypercalcemia, hypercalciuria).^11^, ^29^ Further work is required to elucidate the relationship between calcium phosphate deposition as nephrocalcinosis and calcium oxalate deposition as nephrolithiasis in feline kidneys.

One of the main limitations associated with our study was small sample size, with a total of 11 kidneys stratified across 3 groups on the basis of ultrasound‐diagnosed nephrocalcinosis. The results therefore should be interpreted with caution. In addition, ultrasonography of the kidneys was performed ante‐mortem (as part of a larger prospective study) and μCT and histopathology were carried out on kidneys taken at necropsy, with a median of 167 days between the 2 visits. It is plausible that the extent of nephrocalcinosis could have changed over time, and cats with CKD without ultrasound‐diagnosed nephrocalcinosis at scanning potentially could have developed nephrocalcinosis during the follow‐up period. Two CKD cats (3 kidneys with suspected nephrocalcinosis and 1 kidney with nephrolithiasis) had repeated ultrasonography performed immediately after euthanasia. Comparable ultrasonographic findings regarding the extent of nephrocalcinosis were identified in both scans (8 and 240 days apart, respectively), suggesting minimal changes in nephrocalcinosis during the follow‐up period in these 2 cats. Further study is required to confirm the relationship between ultrasound‐diagnosed nephrocalcinosis and microscopic findings from μCT and histopathology found in our study, especially because this comparison ideally should have been undertaken at the same timepoint (ie, shortly after euthanasia).

In conclusion, our study emphasizes the potential use of μCT to visualize and quantify nephrocalcinosis in feline kidneys, and provides evidence of both macroscopic (μCT) and microscopic nephrocalcinosis (histopathology after von Kossa and Alizarin red staining) in cats with suspected ultrasound‐diagnosed nephrocalcinosis. We also found a strong positive correlation between macroscopic and microscopic nephrocalcinosis in cats with azotemic CKD. However, the clinical relevance of nephrocalcinosis in cats with CKD remains to be determined.

## CONFLICT OF INTEREST DECLARATION

Pak‐Kan Tang received a PhD studentship funded by Royal Canin SAS. Rebecca F. Geddes received funding from Petplan, Royal Canin, a Royal Veterinary College (RVC) Internal Grant, The Academy of Medical Sciences, and The Everycat Foundation; has previously had a consultancy agreement with Boehringer Ingelheim; and has received speaking honoraria from Boehringer Ingelheim, Idexx, and Royal Canin. Rosanne E. Jepson received funding from PetPlan, Feline Foundation for Renal Research, RVC Internal Grant, PetSavers, and has consultancy agreements with Boehringer Ingelheim, Merial, and Ceva Animal Health Ltd, as well as received speaking honoraria from Boehringer Ingelheim, Hills Pet Nutrition, and Ceva Animal Health Ltd. Jonathan Elliott has consultancy agreements with Elanco Ltd, Ceva Animal Health Ltd, Boehringer Ingelheim Ltd, MSD Animal Health Ltd, Orion Incorp, Idexx Ltd, Waltham Petcare Science Institute, Invetx Inc, and Zoetis Ltd and has received grant funding from Elanco Ltd, Waltham Centre for Pet Nutrition, Royal Canin SAS, Idexx Ltd., and Ceva Animal Health Ltd. He is a member of the International Renal Interest Society, which receives sponsorship from Zoetis.

## OFF‐LABEL ANTIMICROBIAL DECLARATION

Authors declare no off‐label use of antimicrobials.

## INSTITUTIONAL ANIMAL CARE AND USE COMMITTEE (IACUC) OR OTHER APPROVAL DECLARATION

This study was part of a larger observational cohort for which approval of the Ethics and Welfare Committee of the Royal Veterinary College (URN 2013 1258E) had been granted.

## HUMAN ETHICS APPROVAL DECLARATION

Authors declare human ethics approval was not needed for this study.

## Supporting information


**Figure S1:** Line graphs illustrating the changes in plasma concentrations of (A) creatinine; (B) symmetric dimethylarginine (SDMA); (C) phosphate; (D) total calcium; and (E) ionized calcium between last ante‐mortem ultrasonography performed and the visit nearest to death in the 7 cats.
