# Supplementary material for: Detection of nephrocalcinosis using ultrasonography, micro‐computed tomography, and histopathology in cats
Source: J Vet Intern Med. 2024 Feb 13;38(3):1553–62. doi: 10.1111/jvim.17011 (PMC11099790; doi:10.1111/jvim.17011)

SUPPLEMENTARY FIGURE 1. Line graphs illustrating the changes in plasma concentrations of (A) creatinine; (B) symmetric dimethylarginine (SDMA); (C) phosphate; (D) total calcium; and (E) ionized calcium between last ante-mortem ultrasonography performed and the visit nearest to death in the 7 cats.

A

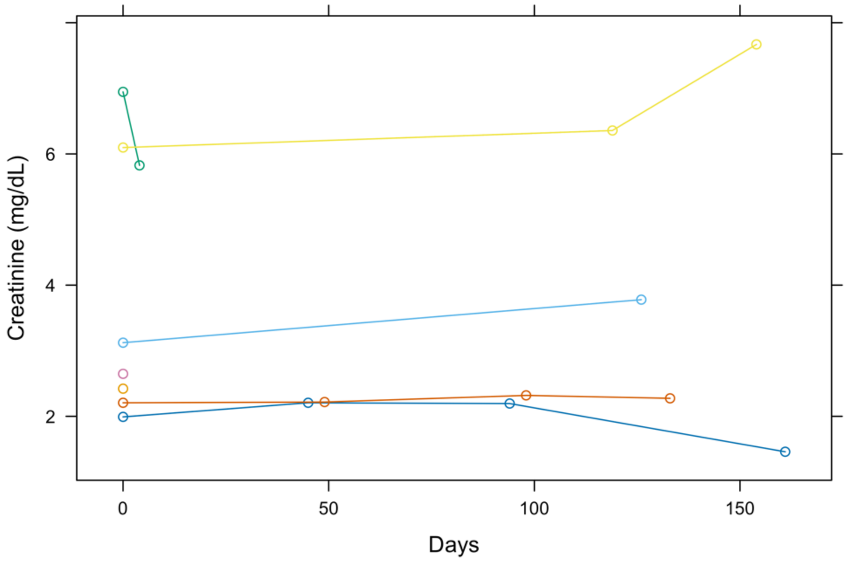

B

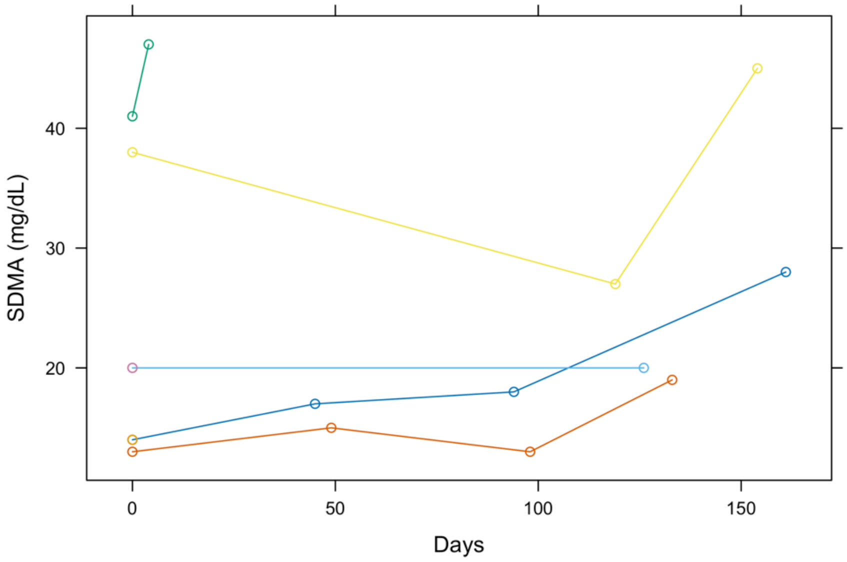

C

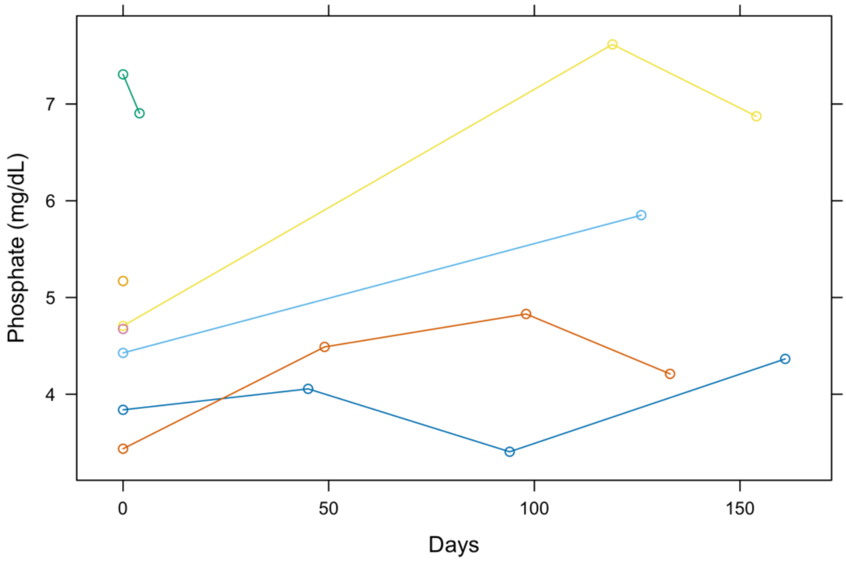

7
6
5
4
Phosphate (mg/dL)
0
50
100
150
Days

D

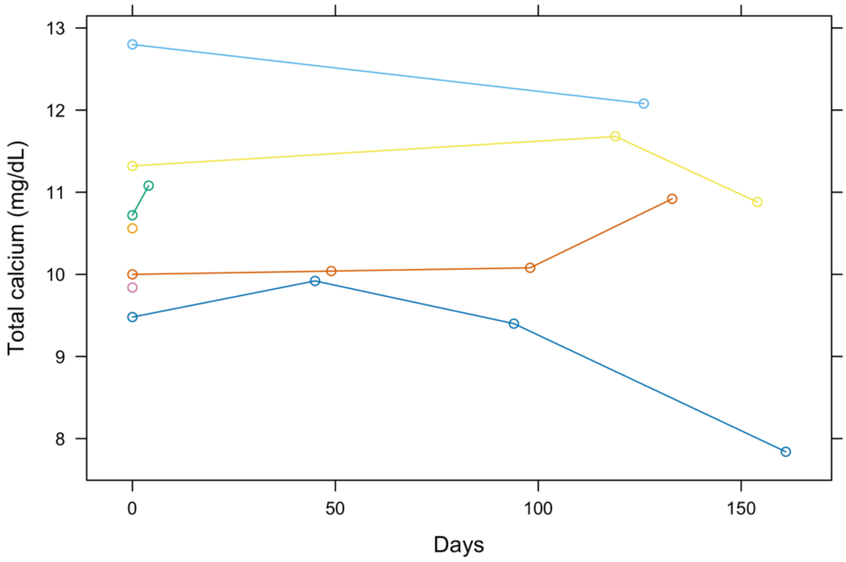

13
12
11
10
9
8
Total calcium (mg/dL)
0
50
100
150
Days

E

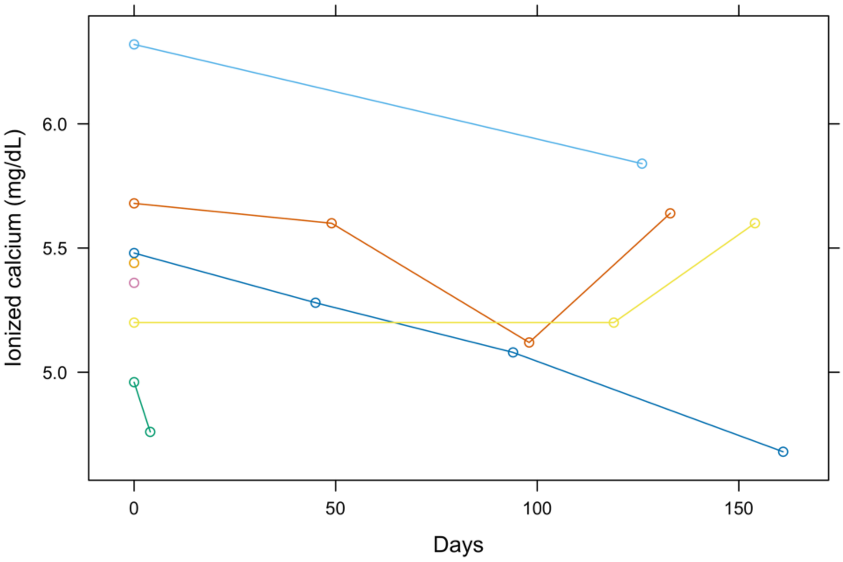

Supplement: Supplementary file 1 — Figure S1: Line graphs illustrating the changes in plasma concentrations of (A) creatinine; (B) symmetric dimethylarginine (SDMA); (C) phosphate; (D) total calcium; and (E) ionized calcium between last ante‐mortem ultrasonography performed and the visit nearest to death in the 7 cats. [file JVIM-38-1553-s001.pdf]
